# Selection of endogenous genes for gene expression studies in *Eucalyptus *under biotic (*Puccinia psidii*) and abiotic (acibenzolar-S-methyl) stresses using RT-qPCR

**DOI:** 10.1186/1756-0500-3-43

**Published:** 2010-02-24

**Authors:** Leonardo P Boava, Marcelo L Laia, Tiago R Jacob, Karina M Dabbas, Janaína F Gonçalves, Jesus A Ferro, Maria IT Ferro, Edson L Furtado

**Affiliations:** 1 Departamento de Produção Vegetal, Setor de Defesa Fitossanitária, Faculdade de Ciências Agronômicas, Universidade Estadual Paulista - UNESP, CEP 18603-970, CP 237, Botucatu, SP, Brazil; 2 Departamento de Tecnologia, Faculdade de Ciências Agrárias e Veterinárias, Universidade Estadual Paulista - UNESP, Via de Acesso Prof. Paulo Donato Castellane, s/n, CEP: 14884-900, Jaboticabal, SP, Brazil; 3 Departamento de Engenharia Florestal, Universidade do Estado de Santa Catarina, Av. Luiz de Camões, 2090, Bairro Conta Dinheiro, CEP 88520-000, Lages, Santa Catarina, Brazil

## Abstract

**Background:**

Rust caused by *Puccinia psidii *Winter has been limiting for the establishment of new *Eucalyptus *plantations, as well as for resprouting of susceptible genetic materials. Identifying host genes involved in defense responses is important to elucidate resistance mechanisms. Reverse transcription-quantitative PCR is the most common method of mRNA quantitation for gene expression analysis. This method generally employs a reference gene as an internal control to normalize results. A good endogenous control transcript shows minimal variation due to experimental conditions.

**Findings:**

We analyzed the expression of 13 genes to identify transcripts with minimal variation in leaves of 60-day-old clonal seedlings of two *Eucalyptus *clones (rust-resistant and susceptible) subjected to biotic (*P. psidii*) and abiotic (acibenzolar-S-methyl, ASM) stresses.

**Conclusions:**

For tissue samples of clones that did not receive any stimulus, a combination of the *eEF2 *and *EglDH *genes was the best control for normalization. When pathogen-inoculated and uninoculated plant samples were compared, *eEF2 *and *UBQ *together were more appropriate as normalizers. In ASM-treated and untreated leaves of both clones, transcripts of the *CYP *and *elF4B *genes combined were the ones with minimal variation. Finally, when comparing expression in both clones for ASM-treated leaves, *P. psidii*-inoculated leaves, ASM-treated plus *P. psidii*-inoculated leaves, and their respective controls, the genes with the most stable expression were *EgIDH *and *UBQ*. The chitinase gene, which is highly expressed in studies on plant resistance to phytopathogens, was used to confirm variation in gene expression due to the treatments.

## Background

Little is known about the genes and metabolic routes involved in defense responses in the genus *Eucalyptus*, whether induced by pathogen infection or induced by treatment with chemical compounds. Therefore, analyzing the behavior of genes likely involved in defense responses may identify means of triggering defense responses that would allow the use of genetic material with good silvicultural characteristics, even if susceptible to a certain disease. To this objective, a great strategy is the analysis of expression of genes likely involved in such mechanisms.

Analysis of gene expression has contributed to a better understanding of plant responses to different stress levels. This genomic approach has led to the identification of many genes induced by stress conditions as well as signaling pathways for genes related to defense in many host-pathogen interactions [1]. On the other hand, gene expression analysis requires sensitive and accurate techniques capable of reproducing the levels of messenger RNA (mRNA) expressed in a cell at a specific moment. Until recently, gene expression levels were generally determined through the northern blotting technique, which is slow, requires microgram amounts of mRNA [2] and poorly detects low expression levels. Currently, RT-qPCR is the most sensitive method for detection of gene expression at both low and high levels. Thus, such a technique can be used for several purposes such as clinical diagnosis, gene expression analysis in a specific tissue, or studies involving complex experiments and a large number of genes [3,4].

To avoid inherent variability, RT-qPCR requires a reference gene with stable expression over the course of the experiment, i.e., a gene with expression levels little influenced by the experimental, developmental or environmental conditions. Studies have shown that several genes considered invariable in terms of expression vary under different experimental conditions [5,3,6]. It is almost impossible to obtain only one invariable gene. According to Vandesompele et al. [7], at least two or three genes should be used as endogenous controls since the use of only one gene for normalization could result in relatively large errors.

Several endogenous controls have been described for the normalization of gene expression through RT-qPCR [3]. However, most of these genes are specific for studies in human tissues [8], viruses and bacteria [9], rice [5], potato [3], *Arabidopsis *[6], *Populus *[10], and sugar cane [11]. Few endogenous genes have been identified so far in many other species; in addition, it is recommended that a group of normalizers be established for each species and that a test be done for each experiment in order to assure their stability in that particular situation.

Thus, this study aimed to analyze the stability of 13 candidate endogenous normalizer genes in *Eucalyptus *subjected to biotic and abiotic stresses. To assure variation among treatments, we also analyzed the expression of the chitinase gene, universally accepted as a gene that responds to infection by phytopathogens or inducers of resistance in resistant plants.

## Methods

### Plant material, resistance inducer treatment and inoculation

Sixty-day-old clonal seedlings from *Eucalyptus grandis *× *Eucalyptus urophylla *Urograndis hybrids were graciously supplied by the Votorantim Celulose e Papel company. Based on resistance tests with the fungus *P. psidii*, carried out at the Phytosanitary Defense Sector, College of Agronomical Sciences (FCA), São Paulo State University (UNESP), Botucatu, São Paulo State, Brazil, as well as on the disease incidence and severity indexes obtained in surveys by the company, we selected two clones: *P. psidii*-resistant (C0) and susceptible (VR).

The resistance inducer acibenzolar-S-methyl (ASM) in the form of the Bion 500 WG commercial product (Syngenta), which is an acetylsalicylic acid analog, was sprayed at a dose of 50 ppm. After five days, leaves were inoculated with *P. psidii*. The inoculum was obtained by collecting *P. psidii *uredospores from naturally infected eucalyptus plants in the field; then, a 5 × 104 spores/ml suspension was prepared in distilled water containing Tween 80 (0.05%). The inoculation method consisted of applying this suspension onto the dorsal surface of leaves with the aid of a vacuum pump. Plants were then kept in a growth chamber at 21°C with a 12 h photoperiod and high relative humidity. Plants presenting the same features served as controls and did not receive any of the treatments (ASM or *P. psidii*). At 24 h after inoculation, leaves were harvested for RNA extraction.

#### Total RNA extraction and cDNA synthesis

RNA was extracted from leaves according to the CTAB buffer method, as described by Chang et al. [12], and quantified in a NanoDrop-ND1000 (Thermo Fisher Scientific Inc.) spectrophotometer at a 1:10 (v/v) dilution.

To a microcentrifuge tube, 4 μg total RNA, 1 μl oligo-dT (500 μg/ml) (dT12-18, Invitrogen), and 1 μl dNTPs (10 mM each), were added to a final volume of 14 μl. The mixture was heated at 65°C for 5 min and then kept on ice. Then, 4 μl first strand buffer (5×, BRL), 2 μl DTT (100 mM), and 1 μl RNAguard (GE Healthcare Life Sciences) were added to the reaction, which was heated at 42°C for 2 min; 1 μl of Superscript II reverse transcriptase (BRL, 200 U/μl) was added, followed by rapid microcentrifugation and incubation at 42°C for 50 min. After this period, the reaction was inactivated at 70°C for 15 min. To remove RNA complementary to the cDNA, 1 μl (2 U) *E. coli *RNase H was added, followed by incubation at 37°C for 20 min. Finally, cDNA samples were purified with a Wizard SV Gel and PCR Clean-Up System kit (Promega) according to the manufacturer's instructions.

#### Primer selection

Based on data available in the literature, 13 genes were selected to test the stability of their expression under biotic and abiotic stresses, as well as one gene of interest for validation (Additional file 1). After this selection, the sequences of the respective genes were recovered from the databank of the *Eucalyptus *Genome Project FORESTs (Eucalyptus Genome Sequencing Project Consortium, https://forests.esalq.usp.br/). Oligonucleotides were generated using Primer Express 2.0 software (Applied Biosystems); the following parameters were specified: length between 20 and 25 bp; melting temperature between 58 and 60°C; GC content between 40 and 60%; and mean length of amplified fragments between 50 and 150 bp. Oligonucleotide synthesis was performed by Integrated DNA Technologies, Inc. (IDT - http://www.idtdna.com).

### Real-time PCR

To detect possible RNA contamination with genomic DNA in leaf samples, PCR using all endogenous candidate genes was carried out with total RNA samples to observe bands indicative of contamination. The reaction consisted of 2.0 μl each primer (forward and reverse) (10 pmol); 2.0 μl 10× PCR buffer; 1.0 μl dNTP mix (10 mM) (Invitrogen); 1.5 μl MgCl2 (25 mM) (Invitrogen); 2.0 μl total RNA (30 ng); 0.25 μl *Taq *polymerase (5 U/μl) (Invitrogen); and 11.25 μl H2O. PCR cycles were: 3 min initial denaturation at 95°C; followed by 35 cycles of 45 s at 94°C, 30 s at 55°C, 1.5 min at 72°C; and a 10 min final extension at 72°C. Amplified products were electrophoresed in an agarose gel at 70 V constant for about 50 min, followed by exposure to UV light in a trans-illuminator; the image was captured for analysis using a CCD camera (Eastman Kodak Company; Rochester, New York). No bands were observed due to primer amplification (data not shown). Using the same reaction conditions, the same number of cycles, and cDNA instead of RNA, we carried out PCR to check the amplicon length. All reaction products showed the expected length (data not shown). From these reactions, amplicons for chitinase, *EgIDH*, and ubiquitin were cloned and sequenced. All of them were confirmed to be the expected amplicon (data not shown).

Real-time reactions used Power SYBR^® ^Green PCR Master Mix and RT-PCR reagent kit (Applied Biosystems). The reaction consisted of 2.0 μl cDNA (30 ng) and a solution containing 2.25 μl DEPC-treated water, 2 μl forward primer (900 nM), 2 μl reverse primer (900 nM), and 6.25 μl SYBR^® ^Green PCR Master Mix. Reactions were performed in duplicate for each sample, including negative controls, in which cDNA was substituted for the same volume of water. An ABI PRISM 7500 Sequence Detection System (Applied Biosystems) was used with the following thermal cycles: one cycle of 50°C for 2 min; one cycle of 95°C for 10 min; 40 cycles of 95°C for 0.15 min and 60°C for 1 min.

### Statistical analysis

Experimental design was completely randomized and included 3 independent biological replicates, in which each plant represented one replicate, in a 2 × 2 × 2 factorial arrangement: 2 clones, resistant and susceptible, sprayed with ASM or distilled water (control), and inoculated with *P. psidii *or uninoculated (control). Leaves were harvested 24 h after inoculation. The expression level and stability of expression of 13 candidate genes were evaluated under these conditions. Expression levels were assessed based on the number of amplification cycles needed to reach a fixed threshold (Cycle threshold - Ct) in the exponential phase of PCR. Statistical analysis was by GenEx version 4.3.6 [13] and geNorm software [7].

## Results

### Stability of endogenous gene expression

**Figure 1:**
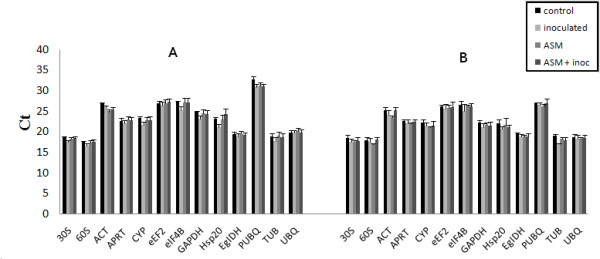
**Expression transcription levels of candidate endogenous control genes**. Expression transcription levels of candidate endogenous control genes and mean Cycle threshold (Ct) in acibenzolar-S-methyl-treated (ASM), *Puccinia psidii*-inoculated (inoculated), and ASM-treated plus *P. psidii*-inoculated (ASM + inoc) *Eucalyptus *clones treatments, and their respective controls. Ct values are the means of 3 biological replicates in duplicate. (a) Clone C0; (b) Clone VR.

To identify the most suitable endogenous control genes, the level of transcript accumulation of the samples was verified with respect to two stress types, biotic (*P. psidii*) and abiotic (ASM), and different genotypes (C0 and VR clones). The expression profile of the 13 genes is shown in Figure 1 and Additional file 2. Genes *30S*, *60S*, *EgIDH*, *TUB *and *UBQ *had the highest expression levels (lowest Ct value) in both clones (Figure 1, Additional file 3). Except for *PUBQ*, the remaining genes did not have substantial differences in Ct value between clones. However, Ct variations due to ASM application and *P. psidii *inoculation were observed at different levels in several genes (Figure 1, Additional file 2).

To choose the best candidate endogenous genes, i.e., the most stably expressed genes following the pathogen and resistance inducer treatments, GeNorm [7] and GenEx version 4.3.6. software [13] were used. Vandesompele et al. [7] defines an expression stability mean value (M-value) as a parameter for quantification of the stability of candidate endogenous genes, in which a low M-value indicates more stable expression, making a certain candidate more appropriate for an endogenous gene control. This value is based on the geometric mean of multiple endogenous genes and the mean variation of a gene relative to all others in a sample group, according to the principle that the relationship between the expression of two ideal endogenous control genes is identical in all samples, and is independently of the experimental conditions.

**Figure 2:**
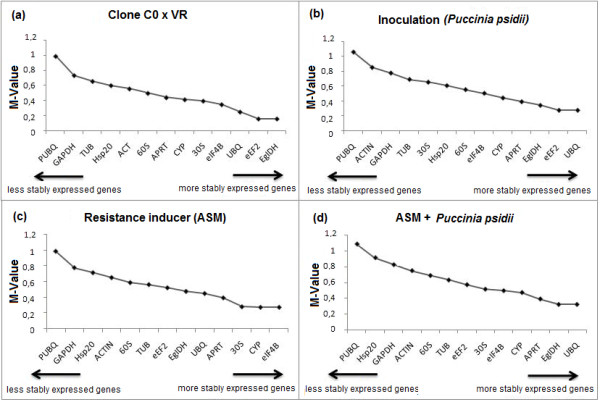
**Expression stability mean values (M-Value) of the 13 candidate endogenous control genes**. Expression stability mean values (M-Value) of the 13 candidate endogenous control genes after analysis using geNorm software. (a) Gene expression stability in tissue samples from *Eucalyptus *C0 and VR clones; (b) gene expression stability in plant samples from *Puccinia psidii*-inoculated and non-inoculated *Eucalyptus *C0 and VR clones; (c) gene expression stability in plant samples from acibenzolar-S-methyl (ASM)-treated and non-treated *Eucalyptus *C0 and VR clones; (d) gene expression stability in plant samples from ASM-treated, *P. psidii*-inoculated, ASM-treated plus *P. psidii*-inoculated *Eucalyptus *C0 and VR clones, and their respective controls, which were neither inoculated with *P. psidii *nor treated with ASM.

In the present work, stability of expression was observed in several situations. Considering only C0 and VR tissue samples that did not receive any stimuli, *eEF2 *and *EgIDH *had the lowest mean values of expression stability (M-value: 0.157), i.e., these genes had more stable expression relative to the other genes evaluated (Figure 2a). Comparison of *P. psidii*-inoculated and uninoculated C0 and VR samples indicated that *eEF2 *and *UBQ *were the most stably expressed genes (M-value: 0.276) (Figure 2b). In ASM-treated and untreated leaves of both clones, *CYP *and *elF4B *had an M-value of 0.275 and were the most stably expressed genes (Figure 2c). Comparing ASM-treated, *P. psidii*-inoculated, and ASM-treated plus *P. psidii*-inoculated leaves of both clones, as well as their respective controls, *EgIDH *and *UBQ *genes had the lowest stability mean values (M-value: 0.321) (Figure 2d). In all cases, *PUBQ *was the least stably expressed gene because it had the highest M-values.

### Chitinase gene relative expression

**Figure 3:**
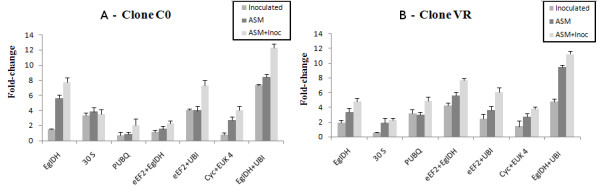
**Relative expression levels of chitinase gene**. Relative expression levels of chitinase gene in acibenzolar-S-methyl-treated (ASM), *Puccinia psidii*-inoculated (inoculated), ASM-treated plus *P. psidii*-inoculated (ASM + inoc) *Eucalyptus *clones, and their respective controls. Data were normalized using genes *EgIDH*, *30S *and *PUBQ *separately and the two most stable candidate endogenous genes associated, considering each experimental variable, according to GeNorm (*eEF2+EgIDH*;*eEF2+UBQ*; and *EgIDH+UBQ*) (Figure 2). (a) Clone C0; (b) Clone VR.

To confirm the stability of candidate endogenous gene expression according to geNorm software and to assure variation in gene expression in response to the experimental conditions (ASM and *P. psidii *treatments), the level of chitinase gene expression was assessed. This gene has been highly expressed in studies related to plant resistance to phytopathogens [14,15] and in response to ASM application [14,1,16]. Chitinase gene expression levels were determined according to the number of amplification cycles needed to reach a fixed threshold (Ct) in the exponential phase of PCR and analyzed using the GenEx version 4.3.6 software. The data were normalized according to the most stable candidate endogenous genes (*eEF2*+*EgIDH*; *eEF2*+*UBQ*; and *EgIDH*+*UBQ*) identified by geNorm software under the various experimental conditions (Figure 2); genes *EgIDH*, *30S *and *PUBQ*, which have been greatly employed as the sole normalizer genes in several studies, were also used. After data normalization with respect to endogenous genes, chitinase gene expression differences were verified among ASM-treated, *P. psidii*-inoculated, ASM-treated plus *P. psidii*-inoculated C0 (Figure 3a) and VR clones, and their respective controls.

The *EgIDH *gene has been employed in several studies as an endogenous reference gene for data normalization [17-19]; however, when this gene was used as the only normalizer for chitinase gene data, the results did not indicate changes in the relative expression of the chitinase gene under any experimental conditions (Figure 3a and 3b). Chitinase gene expression could be differentiated in ASM-treated C0 and VR clones (upregulated 5.58-fold and 3.32-fold, respectively), and ASM-treated plus *P. psidii*-inoculated C0 and VR clones (upregulated 7.70-fold and 4.82-fold, respectively), relative to the respective controls (Additional file 3); however, no difference was observed in *P. psidii*-inoculated C0 and VR clones (upregulated 1.48-fold and 1.87-fold, respectively).

The *30S *gene has also been used in many studies as the only endogenous gene control [3]. When this gene was employed as the only normalizer for chitinase gene relative expression data, increased expression could only be detected in the C0 clone under for *P. psidii*-inoculated (upregulated 3.30-fold), ASM-treated (upregulated 3.81-fold), and ASM-treated plus *P. psidii*-inoculated (upregulated 3.54-fold) leaves (Additional file 3). In the VR clone, only ASM-treated leaves had an increase (upregulated 2.25-fold) in chitinase gene expression (Additional file 3).

The *PUBQ *gene had the highest variation in expression variations across experimental conditions, according to geNorm results (Figure 2). When used for chitinase gene data normalization, it was not useful in detecting changes in level of expression under the different experimental conditions (Figure 3). Only the VR clone had increased expression for *P. psidii*-inoculated (upregulated 3.13-fold), ASM-treated (upregulated 2.96-fold), and ASM-treated plus *P. psidii*-inoculated (upregulated 4.89-fold) leaves, compared with the respective controls (Additional file 3). For the C0 clone, an increase in expression relative to control was only observed in ASM-treated plus *P. psidii*-inoculated leaves (upregulated 2.01-fold).

Comparing tissue samples from C0 and VR clones that did not receive any stimulus, *eEF2 *and *EgIDH *were the most stably expressed genes according to geNorm (Figure 2a). When such genes were used together for chitinase gene data normalization, there was a substantial difference: chitinase expression was higher in the C0 than the VR clone (Figures 3a and 3b). After stimulus, the VR clone had chitinase gene expression increases higher for *P. psidii*-inoculated (upregulated 4.27-fold), ASM-treated (upregulated 5.58-fold), and ASM-treated plus *P. psidii*-inoculated (upregulated 7.70-fold) leaves. However, the C0 clone showed expression differences only for ASM-treated plus *P. psidii*-inoculated (upregulated 2.25-fold) leaves (Additional file 3).

*eEF2 *and *UBQ *were considered the most stably expressed genes in comparisons of expression levels of *P. psidii*-inoculated and uninoculated C0 and VR leaves. When used together for chitinase gene normalization, these genes helped to detect expression differences in both clones with respect to all variables relative to control (Figure 3 and Additional file 3). *Cyc *and *Euk4 *were the most stably expressed genes in the comparison between the expression levels of ASM-treated and untreated leaves. When used together for chitinase gene normalization, these genes only had changes in expression in ASM-treated leaves. The C0 clone had 2.75-fold for ASM-treated leaves and 4.02-fold higher expression for leaves treated both with ASM and inoculated with *P. psidii *relative to controls. VR clone had a similar behavior, upregulated 2.68-fold for ASM-treated leaves and 3.78-fold for ASM-treated plus *P. psidii*-inoculated leaves, relative to controls.

ASM-treated, *P. psidii*-inoculated, and ASM-treated plus *P. psidii*-inoculated clones, *EgIDH *and *UBQ *were the most stably expressed genes (Figure 2d). When used together for chitinase gene normalization, these genes best detected changes in chitinase expression with respect to all variables. In examining the effect of *P. psidii *inoculation, the C0 clone had a 7.33-fold and VR clone had a 4.79-fold increase relative to controls. For the ASM treatment effect, the C0 clone had an 8.41-fold increase in chitinase expression level and the VR clone a 9.45-fold increase. ASM treatment followed by *P. psidii *inoculation 5 days later led to a 12.28-fold increase in expression in the C0 clone and an 11.24-fold increase in the VR clone.

## Discussion

Most of the studied candidate endogenous genes varied due to the experimental conditions (Figure 2), as a function of different genotypes under biotic (*P. psidii*) and abiotic (ASM) stimuli. According to Jain et al. [5], the expression of endogenous genes used for normalization in real-time PCR should be keep constant among cells of different tissues and under different experimental conditions; otherwise, it may lead to incorrect results. For Hendriks-Balk et al. [20], normalization is so routinely used to compare the levels of lipids, proteins or mRNA in two or more sample groups that the underlying assumptions are frequently ignored, which can yield doubtful results and conclusions.

In the literature, most studies on gene expression using RT-qPCR employ only one endogenous gene for data normalization [3]. In *Eucalyptus*, several gene expression studies have adopted only one endogenous reference gene, *EgIDH*, for data normalization [17,18]. To study the transcript levels of several genes in *Eucalyptus *wood formation, Paux et al. [19] employed *EgIDH *as the endogenous reference gene, with relative quantification based on the mathematic model of Pfaffl [21]. In the present work, *EgIDH *was considered the most stably expressed gene (Figure 2); however, it was only efficient in highlighting more intense chitinase expression differences under the experimental conditions contrasting genotypes and biotic (*P. psidii*) and abiotic (ASM) stimuli when associated with *UBQ *for data normalization.

Although ribosomal RNA (rRNA) genes have been widely used as an internal control for pattern of expression [8,22], highly stable expression of such rRNA genes (*30S *and *60S*) was not observed under our experimental conditions (Figure 2), in disagreement with the RT-qPCR results obtained by Zhang & Hu [23] for *Oryzias latipes*, which, under stimulus, had extremely stable ribosomal gene expression. Thellin et al. [24] recommended the use of *28S *and *18S *rRNA as endogenous reference genes in mRNA quantification studies since the variations observed were too weak to modify the total RNA level. However, several authors are against the use of rRNA as endogenous control genes. According to Spanakis [25], ribosomal subunit transcription is affected by biological factors and drugs. For Takle et al. [26], rRNAs are highly conserved in different bacterial species, even inside eukaryotic chloroplasts and mitochondria. Nonspecific amplifications could occur due to plant material, as well as to bacteria naturally present in the phyllosphere. These authors do not recommend the use of a rRNA gene as a reference unless the analysis is of pure cultures. Another disadvantage of rRNA normalization is that cell ribosomal RNA content is much higher than that of mRNA, which requires cDNA samples to be diluted before real-time analysis, likely yielding invalid results [3]. According to Sturzenbaum & Kille [27], some ribosomal subunits do not have poly-A tails, and therefore cannot be amplified from cDNA derived from total RNA using oligo-dT primers in real-time reactions; thus, the use of ribosomal subunit genes has failed to replace other endogenous genes.

Some studies have also used the polyubiquitin gene as an endogenous reference gene. Czechowski et al. [6] observed high expression stability for genes from the polyubiquitin family in several *Arabidopsis *tissues. However, in the present study, *PUBQ *gene had the highest variation in expression under our experimental conditions (Figure 2). Aranda et al. [28] observed high expression of *PUBQ *associated with the viral replication of Pea seed-borne mosaic virus in pea plants. Nogueira et al. [29] also detected high polyubiquitin gene expression when sugarcane plants were subjected to low temperatures, and correlated their estimates of expression to recovery from water stress resulting from exposure to low temperatures.

The most common endogenous genes that have been described for the normalization of expression signals are: actin, glyceraldehyde-3-phosphate dehydrogenase, cyclophilin, elongation factor 1-a (*ef1a*), adenine phosphoribosyl transferase (*aprt*), and tubulin [3]. However, in the present study, GeNorn software did not recommend selecting any of these genes as the sole normalizer. From 13 candidates, we found four pairs that could be used, depending on experimental procedures. According to Vandesompele et al. [7], at least two or three endogenous genes should be used as internal references, since the use of only one gene for normalization can lead to relatively large errors. Jain et al. [5] studied the stability of 10 endogenous genes in different tissue types in rice culture under several abiotic stresses and observed that *UBQ *and *eEF2 *offered the best internal controls for stable expression when used together as normalizers.

Under the present experimental conditions, *EgIDH *and *UBQ*, used together, were the most stably expressed genes to be used as normalizers, and the best differentiating differences in chitinase activity across treatments. The chitinase gene is highly expressed in experiments related to plant resistance to phytopathogens [14,15]. Recent studies have shown that the super-expression of chitinase genes in plants has increased the resistance of the latter to pathogens since this enzyme catalyzes the hydrolysis of chitin polymers, the main component in fungal cell walls, and may possess antimicrobial activity [15]. Several studies in plants have also indicated an increase in chitinase gene expression levels due to ASM application [1,14,16]. The inducer does not act directly on the pathogen nor is it transformed into an antimicrobial agent, but rather sensitizes the plant to activate its defense mechanisms in response to the presence of the pathogen [30]. Such mechanisms can involve enzymes such as chitinase, peroxidase, β-1,3-glucanase, phenylalanine ammonia lyase, and polyphenoloxidase.

After data normalization using the two most stably expressed internal control genes, *EgIDH *and *UBQ*, substantial changes in chitinase gene expression level could be observed. In both clones, gene expression increased after *P. psidii *inoculation. However, such an increase was more intense in the resistant (C0) than in the susceptible clone (VR) (upregulated 7.33-fold and 4.79-fold, respectively) (Additional file 3). These data corroborate the results of surveys on rust incidence and severity indexes done by the company supplying us the plants, based on information on the resistance/susceptibility reaction of clones. The effect of ASM alone was also a determinant of the chitinase gene expression level increase in both clones. This increase may be related to the capacity of ASM to activate plant defense mechanisms. Such induction could be an early trigger of biochemical defense reactions only activated by, for example, the attack of a pathogen. It must be emphasized that plant defense mechanisms, which may appear to be inactive or latent, are activated and expressed after contact with or exposure to an inducer [14].

The highest increases in chitinase gene expression levels were found in leaves previously treated with ASM and inoculated with *P. psidii*, with both clones presenting essentially the same behavior (12.28-fold for C0 and 11.24 higher for VR). This mechanism was triggered and caused higher expression, after the pathogen challenge due to the pre-conditioning induced by exposure to ASM. Acibenzolar-S-methyl is a functional analogue of salicylic acid and a novel plant protection product that mimics the pathogen-host interaction and results in systemic acquired resistance in plants [14]. This pre-conditioning is an important component of induced systemic resistance and is associated with an increase in the capacity to rapidly and effectively activate cell defense responses, which are only induced after contact with the challenging pathogen [30]. When an elicitor is present, changes in plant metabolism are perceptible [14]. However, when induced by an elicitor and then challenged with a pathogen, the plant undergoes more intense metabolic alterations relative to a plant only challenged or only induced, which evidences that, when induced by an elicitor, the plant is more capable of responding to the pathogen presence.

## Conclusions

This study showed that:

1) Considering only C0 and VR tissue samples that did not receive any stimuli, *eEF2 *and *EgIDH * genes had the lowest expression stability mean values (M-value: 0.157);

2) Comparison of *P. psidii*-inoculated and uninoculated C0 and VR samples indicated that *eEF2 *and *UBQ *were the most stable genes (M-value: 0.276);

3) In ASM-treated and untreated plants of both clones, *CYP *and *elF4B *had an M-value of 0.275 and were the most stably expressed genes;

4) Comparing ASM-treated, *P. psidii*-inoculated, ASM-treated plus *P. psidii*-inoculated leaves of both clones, and their respective controls, the *EgIDH *and *UBQ *genes had the lowest stability mean values (M-value: 0.321).

5) In all cases, *PUBQ *was the least stably expressed gene, with the highest M-values.

Therefore, researchers can select appropriate pairs of controls based on their experimental design and tissue.

## Competing interests

The authors declare that they have no competing interests.

## Authors' contributions

LPB, TRJ and MLL did the bibliographic research of the genes used as internal control candidates, built the primers used for the qRT-PCR, and executed the *in silico *analysis of the results. LPB and KMD conducted the RNA extraction, cDNA synthesis, and qRT-PCR reactions. MLL was responsible for general coordination of the study. ELF, JAF and MITF coordinated and oversaw the project. ELF conceived the project. MLL, LPB, JFG and ELF were responsible for most data interpretation and final manuscript elaboration. All authors read and approved the final version of the article.

## Supplementary Material

Additional file 1 **Genes and primers used for RT-qPCR analysis**. Genes and primers used for RT-qPCR analysis. Click here for file

Additional file 2 **Transcription levels of candidate endogenous control genes, mean Cycle threshold value (Ct), and standard deviations of 3 biological replicates in duplicate for clones C0 and VR in acibenzolar-S-methyl-treated (ASM), *Puccinia psidii*-inoculated (inoculated), and ASM-treated plus *P. psidii*-inoculated (ASM + inoc) *Eucalyptus *clones, and their respective controls**. A data table showing the mean Ct and standard deviations for data presented on Figure 1. Click here for file

Additional file 3 **Expression levels of chitinase gene. Data were normalized using the most stable candidate endogenous genes, according to geNorm software. Expression differences in Acibenzolar-S-methyl-treated (ASM), *Puccinia psidii*-inoculated (inoculated), and ASM-treated plus *P. psidii*-inoculated (ASM+Inoc) *Eucalyptus *clones, relative to their respective controls, according to fold-change ≥ 2.0 or ≤ 0.5, up-regulated or down-regulated, respectively**. Expression levels of chitinase gene normalized with most stable candidate genes in different situations. Click here for file
