# Supplementary material for: Selection of endogenous genes for gene expression studies in Eucalyptus under biotic (Puccinia psidii) and abiotic (acibenzolar-S-methyl) stresses using RT-qPCR
Source: BMC Res Notes. 2010 Feb 24;3:43. doi: 10.1186/1756-0500-3-43 (PMC2854107; doi:10.1186/1756-0500-3-43)
Supplement: Additional file 1 — Genes and primers used for RT-qPCR analysis. Genes and primers used for RT-qPCR analysis. [file 1756-0500-3-43-S1.DOC]

Additional file 1. Genes and primers used for RT-qPCR analysis

| **Gene Name** | **Gene Product** | **FOREST's Clone name** | **Primer Sequence**  **Forward/Reverse** | **Amplicon length**  **(bp)** |
| --- | --- | --- | --- | --- |
| *30S* | 30S ribosomal | EGCCFB1221F12.g | GGTGTTGAGAATGCTTTGG/  CATCACTTCCACAATTCTTCC | 148 |
| *60S* | 60S ribosomal | EGEQRT4200C07.g | AAGGGAAGAAAGTTTGAGAGG/  TTTCGACTAGCTTCAGCA | 133 |
| *ACT* | Actin | EGACRT3319G02.g | TAAGCATGACAAGGAACCAG/  TCAGGTCCAAGAAATCGT | 110 |
| *APRT* | Adenine phosphoribosyltransferas | EGCCRT3173G04.g | TTTCTCTTCGTGTCGCTG/  ACGCCATGTGTTGATCTC | 85 |
| *CYP* | Cyclophilin | EGQHLV2242E10.g | AGCCAGTTCTTCATCACC/  TCGGTCTATCATTGTTGTCTG | 132 |
| *eEF2* | Eukaryotic translation elongation factor 2 | EGUTFB1041H07.g | TGAGGTTAATGGGATCGTG/  CAGATTATCGTCATCGACCA | 105 |
| *eIF4B* | Eukaryotic translation initiation factor 4B | EGJFSL4201C05.g | CCCAAATATGAACCGTCCA/  GTTCGATCCATAGCGTCC | 146 |
| *GAPDH* | Glyceraldehyde-3-phosphate dehydrogenase | EGEQRT3002F09.g | TTGGATTTCAGACATAGCCT/  GTGAAAGATGAGTGTTACCC | 58 |
| *Hsp20* | Heat shock protein 20 | EGCCSL1015F10.g | TGTTGAATCTGTGTTCCGT/  TTACATGCATCGACTCGT | 66 |
| *EgIDH* | NADP-isocitrate dehydrogenase | EGEQFB1002G08.g | TGGAACTGTTGAGTCTGG/  TTAGGACCATGAATGAGGAG | 59 |
| *PUBQ* | Polyubiquitin | EGEZLV1202E06.g | ATATCCCAATTAGTGCTGATCC/  GAATCTTTATTTCCACGCAGAG | 57 |
| *TUB* | Tubulin | EGJMRT3143E07.g | GTGACATTCCTCCGACTG/  GCAAGAAAGCCTTCCTG | 123 |
| *UBQ* | Ubiquitin | EGEPFB1249H03.g | GAGGGACATCTATCTCTATGAC/  CAACAGTAAGCACACGAG | 131 |
| *QUI* | Chitinase | EGJELV2266H01.g | AAATCTTCAGCATCGCCA/  CCCAATAGACAGCAATTTCTC | 87 |
