# Supplementary material for: Selection of endogenous genes for gene expression studies in Eucalyptus under biotic (Puccinia psidii) and abiotic (acibenzolar-S-methyl) stresses using RT-qPCR
Source: BMC Res Notes. 2010 Feb 24;3:43. doi: 10.1186/1756-0500-3-43 (PMC2854107; doi:10.1186/1756-0500-3-43)
Supplement: Additional file 2 — Transcription levels of candidate endogenous control genes, mean Cycle threshold value (Ct), and standard deviations of 3 biological replicates in duplicate for clones C0 and VR in acibenzolar-S-methyl-treated (ASM), Puccinia psidii-inoculated (inoculated), and ASM-treated plus P. psidii-inoculated (ASM + inoc) Eucalyptus clones, and their respective controls. A data table showing the mean Ct and standard deviations for data presented on Figure 1. [file 1756-0500-3-43-S2.DOC]

Additional file 2: Transcription levels of candidate endogenous control genes, mean Cycle threshold value (Ct), and standard deviations of 3 biological replicates in duplicate for clones C0 and VR in Acibenzolar-S-methyl-treated (ASM), *Puccinia* *psidii*-inoculated (inoculated), and ASM-treated plus *P. psidii*-inoculated (ASM + inoc) *Eucalyptus* clones, and their respective controls.

| **Genes** | Clone C0 | | | | Clone VR | | | |
| --- | --- | --- | --- | --- | --- | --- | --- | --- |
|  | **control** | **inoculated** | **ASM** | **ASM + inoc** | **control** | **inoculated** | **ASM** | **ASM + inoc** |
| *30S* | 18.8 ± 0.19 | 17.6 ± 0.27 | 18.4 ± 0.29 | 18.5 ± 0.36 | 18.4 ± 0.73 | 17.5 ± 0.71 | 17.7 ± 0.31 | 17.7 ± 0.97 |
| *60S* | 17.6 ± 0.14 | 16.6 ± 0.47 | 17.3 ± 0.58 | 17.6 ± 0.51 | 18.0 ± 0.70 | 17.6 ± 0.89 | 16.9 ± 0.25 | 18.1 ± 0.54 |
| *ACTIN* | 26.9 ± 0.15 | 25.9 ± 0.40 | 25.0 ± 0.48 | 25.4 ± 0.57 | 25.1 ± 0.81 | 24.2 ± 1.03 | 23.6 ± 0.45 | 25.2 ± 0.80 |
| *Adenine* | 22.7 ± 0.66 | 22.0 ± 0.81 | 23.0 ± 0.84 | 22.8 ± 0.69 | 22.6 ± 0.48 | 22.0 ± 0.91 | 22.0 ± 0.27 | 22.5 ± 0.56 |
| *Cyclophilin* | 23.4 ± 0.41 | 21.7 ± 0.72 | 22.7 ± 0.93 | 22.9 ± 0.86 | 22.2 ± 0.91 | 21.5 ± 0.72 | 21.2 ± 0.16 | 21.6 ± 0.98 |
| *Eukaryotic 2* | 26.9 ± 0.61 | 26.4 ± 0.91 | 26.9 ± 0.93 | 27.2 ± 0.80 | 26.1 ± 0.40 | 25.7 ± 1.08 | 25.8 ± 0.81 | 25.9 ± 1.45 |
| *Eukaryotic 4B* | 27.2 ± 0.28 | 25.3 ± 0.85 | 27.1 ± 1.16 | 27.1 ± 1.09 | 26.5 ± 1.00 | 25.1 ± 1.57 | 26.3 ± 0.19 | 26.1 ± 0.77 |
| *GAPDH* | 24.8 ± 0.15 | 23.2 ± 0.81 | 24.5 ± 1.00 | 24.2 ± 0.98 | 22.2 ± 0.54 | 21.0 ± 1.05 | 21.8 ± 0.46 | 21.5 ± 1.02 |
| *Hsp20* | 23.2 ± 0.24 | 21.1 ± 0.90 | 23.0 ± 1.22 | 24.4 ± 1.20 | 22.1 ± 0.87 | 20.8 ± 0.49 | 21.8 ± 1.66 | 21.1 ± 0.55 |
| *NADP* | 19.5 ± 0.50 | 18.9 ± 0.67 | 19.5 ± 0.73 | 19.3 ± 0.56 | 19.6 ± 0.22 | 19.0 ± 0.45 | 18.9 ± 0.34 | 18.8 ± 0.85 |
| *Polyubiquitin* | 32.7 ± 0.69 | 30.9 ± 0.70 | 31.2 ± 0.79 | 31.0 ± 0.73 | 27.0 ± 0.24 | 26.5 ± 0.58 | 26.2 ± 0.64 | 27.0 ± 1.13 |
| *Tubulin* | 18.8 ± 0.88 | 17.7 ± 1.02 | 19.0 ± 1.08 | 18.7 ± 0.97 | 19.1 ± 0.34 | 17.1 ± 0.16 | 18.3 ± 0.41 | 17.9 ± 0.73 |
| *Ubiquitin* | 19.9 ± 0.51 | 19.8 ± 0.65 | 20.2 ± 0.83 | 19.9 ± 0.67 | 18.8 ± 0.64 | 18.4 ± 0.80 | 18.4 ± 0.27 | 18.6 ± 0.71 |
