# Supplementary material for: Selection of endogenous genes for gene expression studies in Eucalyptus under biotic (Puccinia psidii) and abiotic (acibenzolar-S-methyl) stresses using RT-qPCR
Source: BMC Res Notes. 2010 Feb 24;3:43. doi: 10.1186/1756-0500-3-43 (PMC2854107; doi:10.1186/1756-0500-3-43)
Supplement: Additional file 3 — Expression levels of chitinase gene. Data were normalized using the most stable candidate endogenous genes, according to geNorm software. Expression differences in Acibenzolar-S-methyl-treated (ASM), Puccinia psidii-inoculated (inoculated), and ASM-treated plus P. psidii-inoculated (ASM+Inoc) Eucalyptus clones, relative to their respective controls, according to fold-change ≥ 2.0 or ≤ 0.5, up-regulated or down-regulated, respectively. Expression levels of chitinase gene normalized with most stable candidate genes in different situations. [file 1756-0500-3-43-S3.DOC]

Additional file 3. Expression levels of chitinase gene. Data were normalized using the most stable candidate endogenous genes, according to geNorm software. Expression differences in Acibenzolar-S-methyl-treated (ASM), *Puccinia psidii*-inoculated (inoculated), and ASM-treated plus *P. psidii*-inoculated (ASM+Inoc) *Eucalyptus* clones, relative to their respective controls, according to fold-change > 2.0 or < 0.5, up-regulated or down-regulated, respectively

| Treat. | Fold-change C0 clone treatment / control | | | | | | |  | Fold-change VR clone treatment / control | | | | | | |
| --- | --- | --- | --- | --- | --- | --- | --- | --- | --- | --- | --- | --- | --- | --- | --- |
|  | EgIDH | 30 S | PUBQ | eEF2  EgIDH | eEF2  UBQ | Cyc  Euk4 | EgIDH  UBQ |  | EgIDH | 30 S | PUBQ | eEF2  EgIDH | eEF2  UBQ | Cyc  Euk4 | EgIDH  UBQ |
| Inoculated | 1.48 | 3.30 | 0.74 | 1.18 | 4.04 | 0.79 | 7.33 |  | 1.87 | 0.54 | 3.13 | 4.27 | 2.40 | 1.40 | 4.79 |
| ASM | 5.58 | 3.81 | 0.86 | 1.54 | 4.00 | 2.75 | 8.41 |  | 3.32 | 1.92 | 2.96 | 5.58 | 3.65 | 2.68 | 9.45 |
| ASM+Inoc | 7.70 | 3.54 | 2.01 | 2.25 | 7.28 | 4.02 | 12.28 |  | 4.82 | 2.25 | 4.89 | 7.70 | 6.07 | 3.78 | 11.24 |
